# Prognostic significance of XIAP and NF-κB expression in esophageal carcinoma with postoperative radiotherapy

**DOI:** 10.1186/1477-7819-11-288

**Published:** 2013-11-05

**Authors:** Suna Zhou, Wenguang Ye, Qiuju Shao, Yuhong Qi, Mingxin Zhang, Jun Liang

**Affiliations:** 1 Department of Radiotherapy, Tangdu Hospital, Fourth Military Medical University, Xinsi Road 1, Xi’an, Shaanxi, China; 2 Department of Gastroenterology, Tangdu Hospital, Fourth Military Medical University, Xinsi Road 1, Xi’an, Shaanxi, China

**Keywords:** Esophageal squamous cell carcinoma, X-chromosome-linked IAP, Nuclear factor-κB

## Abstract

**Background:**

X-chromosome-linked IAP (XIAP) and nuclear factor-κB (NF-κB) are frequently overexpressed and correlate closely with chemoradiotherapy resistance and poor prognosis in many cancers. However, the significance of XIAP and NF-κB expression in radiotherapy sensitivity and its effect on the prognosis of esophageal squamous cell carcinoma (ESCC) are still unknown. The aim of this study was to examine XIAP and NF-κB status in ESCC patients undergoing postoperative radiotherapy after radical surgery, and to evaluate their clinical significance.

**Methods:**

A total of 78 ESCC patients treated with postoperative radiotherapy after radical surgery were enrolled in this study. We immunohistochemically investigated the expression of XIAP and NF-κB in tissues from enrolled patients with specific antibodies. Then, the correlations among XIAP, NF-κB expression, clinicopathological features and its prognostic relevance in ESCC were analyzed.

**Results:**

The increased expression of XIAP and NF-κB in ESCC tissues were clearly correlated with the tumor differentiation and p-TNM stage. Significant positive correlations were found between the expression status of XIAP and NF-κB (r = 0.779, *P* = 0.000). Overexpression of XIAP and NF-κB and metastasis were significantly associated with shorter overall survival times in univariate analysis (*P* < 0.05). Multivariate analysis also confirmed that XIAP expression was an independent prognostic factor (*P* = 0.005).

**Conclusions:**

XIAP and NF-κB are intensively expressed in ESCC. The level of XIAP is positively correlated to progression and prognosis of ESCC.

## Background

Esophageal cancer is one of the most aggressive and lethal malignancies, and esophageal squamous cell carcinoma (ESCC) is the major histologic form of esophageal cancer [1]. Surgery is still the mainstay treatment for patients with esophageal cancer, however, the five-year risk of the operable recurrence is 70% to 80% [2,3]. Postoperative radiotherapy as an adjuvant therapy is being used more often to improve the outcome of ESCC patients after surgery [2]. However, not all patients with ESCC benefit from radiotherapy, and there are individual differences in response to postoperative radiotherapy. The outcomes vary greatly and unpredictably, and the survival of responders has been reported to be better than that of non-responders [4,5]. It is important to recognize the probable treatment response in these tumors. The main obstacle to this approach is the lack of availability of prognostic biomarkers. Pretreatment clinical parameters such as gender, age, TNM classification, and tumor differentiation are not effective in predicting the biologic behavior of ESCC patients who receive postoperative radiotherapy. Thus, it is necessary to identify available biomarkers for predicting response and treatment outcomes of postoperative radiotherapy in ESCC patients.

Among the molecular pathways potentially involved in generating the differential response to radiotherapy, an association has consistently been observed between the apoptotic pathway and the tumor radiosensitivity [4,5]. Inhibitor of apoptosis (IAP) proteins are a family of endogenous antiapoptotic proteins [6]. Among the eight human IAP proteins, X-chromosome-linked IAP (XIAP) has been reported to exert the most pronounced antiapoptotic function, which has been linked to its ability to bind to caspase-3, -7 and -9 [7]. Firstly, a number of studies have demonstrated that elevated expression levels of XIAP in many types of tumors correlates with a poor prognosis [8]. Secondly, both *in vitro* and *in vivo* studies have further demonstrated that down-regulation of XIAP expression, either by RNA interference (RNAi) or antisense oligonucleotides, results in stimulation of sensitization to gamma-irradiation and chemotherapeutic-induced apoptosis in tumor cells [9-12]. Likewise, XIAP has been found to be highly expressed in ESCC, and its downregulation by RNAi sensitizes ESCC cell lines to chemotherapeutics [13]. Additionally, more and more research has shown that XIAP acts as a radioresistance factor for radiotherapy in human cancers [14-19]. However, whether XIAP can play a role as a prognostic marker for radiotherapy in ESCC patients has not been extensively investigated to date.

Moreover, aberrant nuclear factor-κB (NF-κB) expression has been detected in many human malignancies. NF-κB is a transcription factor that regulates the expression of genes linked to inflammation, apoptosis, survival, proliferation, invasion, angiogenesis, metastasis, chemoresistance, tumor cell transformation, and radioresistance [20]. NF-κB may activate the expression of several genes or proteins that are involved in the apoptotic regulation, such as IAPs [21]. Furthermore, XIAP has also been implicated in the regulation of NF-κB activation [22]. On the other hand, NF-κB may be responsible for blocking the efficacy of chemotherapy and radiation in some types of tumor cells. The positive correlation between NF-κB expression in ESCC and their resistance to chemoradiation therapy has been previously reported [23], but more specific studies are required to confirm the significance of NF-κB in predicting disease progression in postoperative radiotherapy of ESCC.

The aim of this study was to determine the prognostic significance of XIAP and NF-κB in terms of overall survival in ESCC treated with surgery followed by radiotherapy. This was done by using immunohistochemical staining to explore the potential markers in 78 ESCC patients who underwent a surgical resection and postoperative radiotherapy. We also investigated whether the expression levels of XIAP correlate with that of NF-κB in this patient population.

## Methods

### Patients and specimens

A total of 88 patients with ESCC were selected for this study between January 2000 and December 2007 in the Tangdu Hospital of Fourth Military Medical University and First Affiliated Hospital of Medical School of Xi’an Jiaotong University. Of these, the tumor staging, clinicopathological information, or follow-up was incomplete for ten patients. As a result, 78 patients were retrospectively reviewed. In addition, patients were required to meet the following criteria: (1) all ESCC cases were pathology confirmed; (2) no distant metastasis (except to the supraclavicular and celiac lymph nodes); (3) the patients completed the whole course of radiotherapy; (4) the patients received preoperative radiotherapy or chemotherapy were excluded; (5) the patients received postoperative chemotherapy or postoperative concurrent chemoradiation were excluded. Tissue samples collected during biopsy and surgery were formalin fixed and paraffin embedded. The Institutional Ethics Committee approval for this study had been obtained from the Tangdu hospital Institutional Review Board.

### Surgery

All patients underwent radical surgery. The surgical approach consisted of a limited thoracotomy on the right side and intrathoracic gastric tube reconstruction (Ivor-Lewis procedure) for lesions in the middle/lower-third of the esophagus. Upper-third lesions were treated by neck anastomosis (Mckeown procedure). Patients underwent two-field or three-field lymph node dissection (the neck, mediastinum and abdomen) depending on the surgical approach used.

### Postoperative radiotherapy

The selection of postoperative adjuvant therapy was made according to the individual physicians’ preference and the general physical conditions of the patient. Postoperative radiation was begun three to four weeks after the surgery. All patients were treated using three-dimensional conformal radiation therapy (3D-CRT) after esophagectomy. The initial treatment volume included the primary tumor and enlarged lymph nodes. The median radiation dose of 48 Gy (40 to 50 Gy, 2 Gy per fraction, five days per week) was delivered with a three- or four-field technique in 20 to 25 fractions. The extent of the irradiation field was determined based on the primary site in the esophagus. For the lesions of the upper/middle-third of the esophagus, the irradiation area included the tumor bed, bilateral supraclavicular fossae, mediastinum, and subcarinal area; for the lower-third lesions of the esophagus, the tumor bed, bilateral supraclavicular fossae, mediastinum, subcarinal area, and lower thoracic paraesophageal lymph nodes area were irradiated.

### Follow-up

Follow-up evaluations were performed every three to four months after radiotherapy until the study was finished or the patient died. The mean duration of follow-up was 23.63 months (±13.14 months).

### Immunohistochemical staining

Tissue specimens were fixed in neutral buffered formalin (10% v/v formalin in water; pH 7.4) and embedded in paraffin wax. Serial sections of 4 μm thickness were cut and mounted on charged glass slides. The monoclonal antibody against XIAP (1:100; Cell Signaling Technology, Beverly, MA, USA) and NF-κB (1:200; Santa Cruz Biotechnology, Santa Cruz, CA, USA) were used respectively. The streptavidin-peroxidase technique (Golden Bridge International: SP-9000, Mukilteo, Washington, USA) was used according the manufacturer's instruction. An irrelevant rabbit antiserum served as a negative control. Sections were counterstained with Mayer’s hematoxylin.

### Immunohistochemical analysis

Two observers who were blinded to clinical and follow-up data evaluated staining results independently and co-observed for a consensus when they were divergent. Both the percentage of positive cells and the strength of the staining were considered in the following method. Five degree magnification visions were chosen randomly under the optical microscope, the calculation of results being as followed: the percentage of positive cells in 0% to 5% was counted 0; the percentage of positive cells in 5% to 25% was counted 1; 26% to 50% was counted 2; 51% to 75% was counted 3; ≥ 76% was counted 4. In respect of staining strength, the score for tumor cells without stain is 0; straw yellow for 1; brown for 2; tan for 3. The staining index score was the sum of the items above. For the purpose of statistical analysis, 3 was used as a cutoff value to distinguish tumors with a low (< 3) or high (≥ 3) level of expression.

### Statistical analysis

Data were analyzed using SPSS version 13.0 (SPSS Institute, Chicago, IL, USA). Fisher’s exact test was used to analyze the correlation between staining index and other categorical factors potentially predictive of prognosis. The Spearman's rank correlation coefficient was used for analyzing the association of NF-κB expression levels with XIAP expression status. Overall survival was determined as the time (in months) from the date of surgery to last follow-up or to 1 January 2013, for living patients or to the date of death. Survival curve and median survival were estimated by the Kaplan-Meier method, and the statistical differences between survival curves were examined by the log-rank test. A Cox proportional hazard regression model was used to perform multivariate analyses. *P* < 0.05 was regarded as statistically significant.

## Results

### Expression of XIAP and NF-κB in ESCC and their relationships to clinicopathological variables

**Figure 1:**
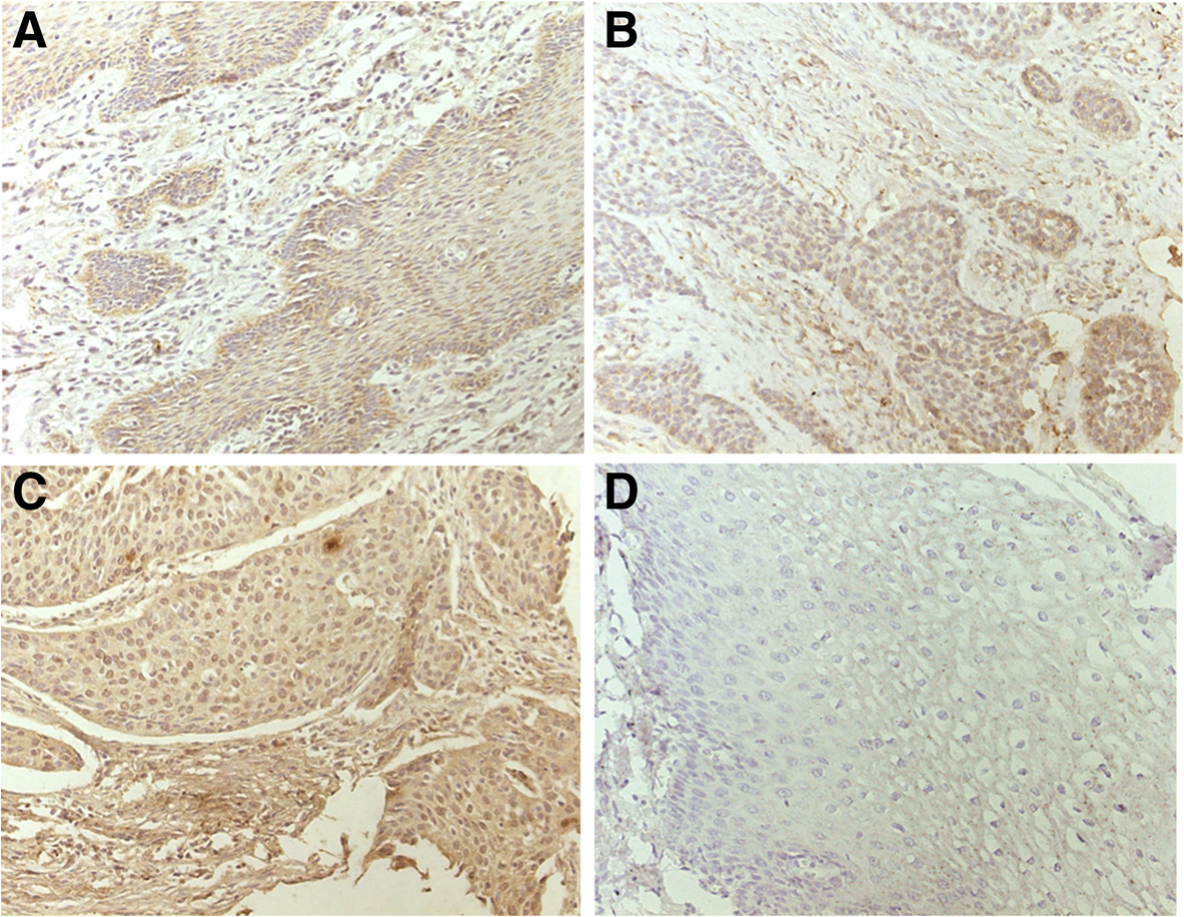
**Immunohistochemical expression of X-linked-chromosome inhibitor of apoptosis (XIAP) protein and nuclear factor-κB (NF-κB) in esophageal squamous cell carcinoma (ESCC). (A)** typical immunohistological features with high levels of XIAP expression in ESCC. The XIAP staining shown cytoplasmic localization; **(B)** typical immunohistological features with lower levels of XIAP expression in ESCC; **(C)** typical immunohistological features with high levels of NF-κB in ESCC. The NF-κB staining was present in the nucleus and cytoplasm of tumor cells; **(D)** negative staining in ESCC. Magnifications × 200.

Levels of XIAP and NF-κB were evaluated by immunohistochemical analysis. XIAP immunoreactivity showed cytoplasmic localization, while NF-κB was mainly located in the nucleus and cytoplasm. Figure 1 shows representative expression patterns of XIAP and NF-κB in ESCC. The increased expression of XIAP and NF-κB in ESCC tissues showed obvious correlation with the tumor differentiation and p-TNM stage (Table 1).

**Table 1:** Correlation of X-linked-chromosome inhibitor of apoptosis (XIAP) protein and nuclear factor-κB (NF-κB) expression to clinicopathologic characteristics of 78 patients with esophageal squamous cell carcinoma (ESCC)

**Variable**	**XIAP expression (case)**			**NF-κB expression (case)**		
	**(case)**			**(case)**		
	**Low**	**High**	***P-*** **value**	**Low**	**High**	***P-*** **value**
Gender						
Male	15	33	0.800	12	36	1.000
Female	8	22		8	22	
Age						
< 60	13	35	0.615	13	35	0.794
≥ 60	10	20		7	23	
Tumor location						
Lower thoracic	14	30	0.628	10	34	0.603
Middle and Upper thoracic	9	25		10	24	
Length of tumor						
< 5 cm	14	30	0.628	10	34	0.603
≥ 5 cm	9	25		10	24	
T-stage						
T_3_	12	41	0.066	14	39	1.000
T_1-2_	11	14		6	19	
N-stage						
N_0_	11	22	0.618	11	22	0.201
N_1_	12	33		9	36	
p-TNM stage						
I-II	13	15	0.020	12	16	0.014
III	10	40		8	42	
Differentiation						
Well and Medium	23	28	0.000	20	31	0.000
Poor	0	27		0	27	

### Association of XIAP expression levels with NF-κB expression status

Since XIAP can activate the transcription factor NF-κB, a known survival factor for cancer cells, we next investigated the association of XIAP expression levels with NF-κB expression status (Table 2). Of the 54 tumors containing a high level of cytoplasmic XIAP immunoreactivity, a total of 51 cases displayed a high expression of NF-κB. We calculated the Spearman's rank correlation coefficient to evaluate the linear relationship. There was statistically significant association of XIAP expression status with NF-κB expression levels (r = 0.779, *P* = 0.000).

**Table 2:** Association of nuclear factor-κB (NF-κB) expression levels with X-linked-chromosome inhibitor of apoptosis (XIAP) protein expression status

**Variables**	**Total**	**XIAP**		***P***	**r**
		**Low**	**High**		
NF-κB				0.000	0.779
Low	20	18	2		
High	58	5	53		

### Survival analysis

**Figure 2:**
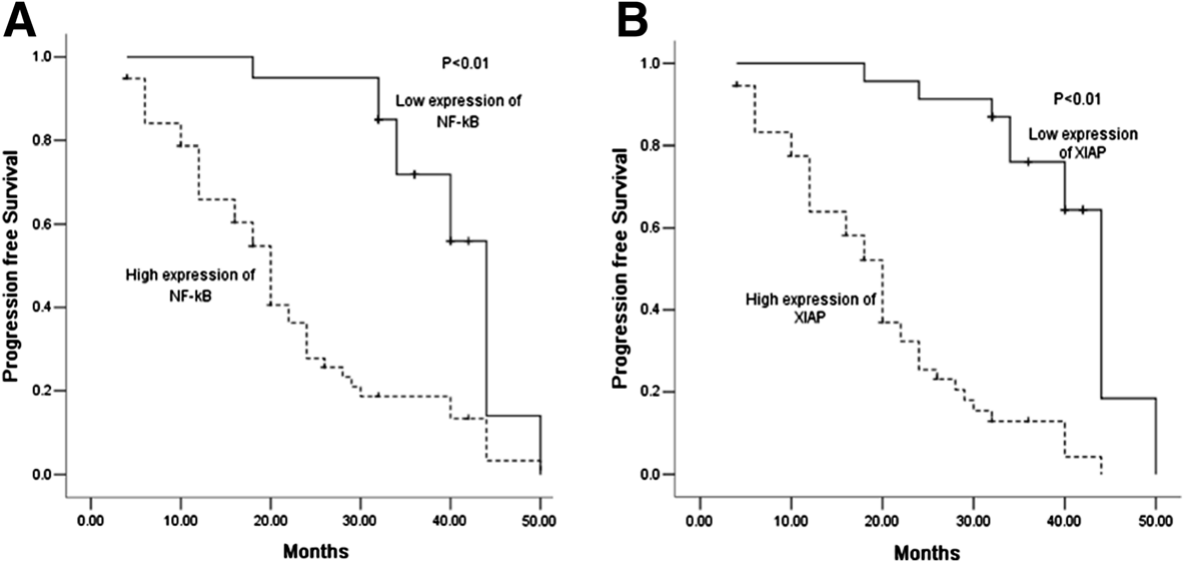
**Kaplan-Meier survival analysis.** Progression free survival differences between patients with high and low levels of protein expression or metastasis. *P-*value was obtained using the log-rank test of the difference. **(A)** X-linked-chromosome inhibitor of apoptosis (XIAP) protein; **(B)** Nuclear factor- κB (NF-κB).

Kaplan-Meier analysis was used to calculate the impact of classic clinicopathologic features and protein expression on survival (Table 3, Figure 2). Differentiation and high expression of XIAP and NF-κB were associated with decreased survival (*P* < 0.05), whereas other clinicopathological variables were not significant. Cox regression analysis revealed a statistically significant correlation among differentiation and XIAP expression (*P* < 0.05, Table 4).

**Table 3:** Univariate analysis for overall survival

**Variables**	**Total**	**Overall survival**		***P***
		**Median ± SE**	**95% CI**	
XIAP				0.000
Low	23	44.00 ± 0.99	42.05 to 45.95	
High	55	20.00 ± 1.30	17.45 to 22.55	
NF-κB				0.000
Low	20	44.00 ± 1.20	41.66 to 46.34	
High	58	20.00 ± 1.37	17.32 to 22.68	
Gender				0.624
Male	48	22.00 ± 1.79	18.48 to 25.52	
Female	30	30.00 ± 4.71	20.77 to 39.23	
Age				0.956
< 60	48	22.00 ± 3.10	15.92 to 28.08	
≥ 60	30	26.00 ± 5.11	15.98 to 36.02	
Tumor location				0.421
Lower thoracic	44	22.00 ± 7.37	7.56 to 36.44	
Middle and Upper thoracic	34	40.13 ± 5.68	19.58 to 28.42	
Length of tumor				0.421
< 5 cm	44	24.00 ± 2.25	19.58 to 28.42	
≥ 5 cm	34	22.00 ± 7.37	7.56 to 36.44	
T-stage				0.202
T_3_	53	24.00 ± 2.59	18.93 to 29.07	
T_1-2_	25	29.00 ± 5.34	18.53 to 39.47	
N-stage				0.238
N_0_	33	24.00 ± 7.65	9.01 to 38.99	
N_1_	45	24.00 ± 2.93	18.25 to 29.75	
p-TNM stage				0.076
I-II	28	32.00 ± 2.82	26.48 to 37.52	
III	50	20.00 ± 2.18	15.74 to 24.26	
Differentiation				0.000
Well and Medium	51	34.00 ± 3.40	27.35 to 40.66	
Poor	27	16.00 ± 2.43	11.24 to 20.76	

**Table 4:** Multivariate Cox proportional hazards analysis for overall survival

**Variables**	**Overall survival**		***P***
	**RR**	**95% CI**	
XIAP	4.68	1.60 to 13.67	0.005
Differentiation	3.55	1.51 to 8.37	0.004

## Discussion

Compared with the surgery alone, earlier studies have suggested that postoperative prophylactic radiotherapy produces a better prognosis in esophageal cancer patients [24-26]. However, not all patients benefit from radiotherapy; some even have a shorter survival time. A good tumor response is usually responsible for an excellent prognosis. Thus, biomarkers that can predict radiation response and outcomes before treatment are needed and a high level of apoptotic tumor cells is a predictive marker for tumor response in antitumor therapy. Among the numerous proteins involved in apoptosis regulation, IAP proteins play an important role. The human IAP family is composed of eight proteins: NAIP (BIRC1), c-IAP1 (BIRC2), c-IAP2 (BIRC3), XIAP (BIRC4), survivin (BIRC5), Apollon/Bruce (BIRC6), ML-IAP (BIRC7 or livin) and ILP-2 (BIRC8). Furthermore, XIAP is the only member of this family able to directly inhibit both the initiation and execution phase of the caspase cascade which is crucial to mediate the controlled demise of malignant cells. More widely, XIAP has been found to be frequently overexpressed in most human cancer cells and renders cells resistant to cancer treatment and so XIAP has received interest as a therapeutic target [9,27]. Recently, increasingly more research studies have found that small-molecule XIAP inhibitors could enhance irradiation-induced apoptosis in most cancer cells [28-30]. However, the prognosis of XIAP for postoperative prophylactic radiotherapy in ESCC patients has not been extensively investigated. On the other hand, the most important contribution of IAPs to cell survival and tumorigenesis resides in the ability of XIAP to regulate ubiquitin-dependent activation of NF-κB [31]. The high constitutive nuclear activation of NF-κB activation has been detected in many solid cancers, which attribute mainly to the development and progression of cancer such as proliferation, migration and apoptosis [21]. A positive correlation was observed between NF-κB and nodal metastasis in ESCC, but the clinical significance for NF-κB in predicting associated outcomes and its correlation with XIAP in patients receiving postoperative radiotherapy for ESCC have not been reported before.

In this study, we found that XIAP and NF-κB were highly expressed in ESCC specimens. Immunostaining showed the expression of XIAP in the resected ESCC specimens to be mostly in the cytoplasm, whereas NF-κB expression was predominantly localized in the nucleus (Figure 1). Consistently, our results showed that the high level of XIAP expression correlated significantly with both tumor differentiation and p-TNM stage, and the same results were obtained for NF-κB. Next, the statistical analysis shows that the *in vivo* correlation between XIAP and NF-κB expression is a highly significant association. This means NF-κB may be one of XIAP-related interacting partners. However, the exact molecular basis by which XIAP might interact with NF-κB remains to be clarified *in vivo*. Overall, the available data so far suggest that XIAP pathway may well also be related to the genetic changes implicated in ESCC radiosensitivity.

Previous investigation has largely focused on the prognostic value of NF-κB in solid cancer, but there have been few reports about that of XIAP [19,32,33]. Moreover, the prognostic value of XIAP for radiotherapy in ESCC is not clear. Based on our observation of 78 patients with ESCC, our study explores the hypothesis that overexpression of XIAP and NF-κB is predictive of shorter survival in ESCC patients following surgery and postoperative. It is generally considered that patients will benefit greatly from antitumor therapy if available accurate information about the likely outcomes before treatment is started can be obtained. Consequently, further study is needed to elucidate markers or combinations of markers which best reflect the effects of radiotherapy in ESCC. Such markers might prove valuable not only as clinical predictors, but also as targets for ESCC treatment: for example, treatment might result in increased sensitivity if these abnormalities of function and expression return to normal.

## Conclusion

In conclusion, high level of XIAP expression was an independent unfavorable prognostic indicator in ESCC patients treated with radiotherapy after surgery. This might be useful in helping clinicians to choose, with more accuracy, the best clinical policy and therapy in patients. Furthermore, we will be able to focus in future on both the prognostic and treatment value of XIAP.

## Abbreviations

XIAP: X-chromosome-linked IAP; NF-κB: Nuclear factor-κB; ESCC: Esophageal squamous cell carcinoma; RNAi: RNA interference; 3D-CRT: Three-dimensional conformal radiation therapy; RR: Relative risk.

## Competing interests

The authors declare that they have no competing interests.

## Authors’ contributions

SZ analyzed the data and wrote the manuscript. MZ and JL commented on and revised the manuscript. WY, QS and YQ built the patient database. All authors read and approved the final manuscript.
